# Telomere length is an epigenetic trait – Implications for the use of telomerase-deficient organisms to model human disease

**DOI:** 10.1242/dmm.050581

**Published:** 2024-03-05

**Authors:** Catarina M. Henriques, Miguel Godinho Ferreira

**Affiliations:** ^1^ The Bateson Centre, MRC-Arthritis Research UK Centre for Integrated Research Into Musculoskeletal Ageing (CIMA) and Healthy Lifespan Institute (HELSI), School of Medicine and Population Health, University of Sheffield, Sheffield S10 2TN, UK; ^2^ Institute for Research on Cancer and Aging of Nice (IRCAN), CNRS UMR7284, INSERM U1081, Université Côte d‘Azur, 06107 Nice, France

**Keywords:** Telomere length, Telomerase, Epigenetic inheritance, Ageing, Zebrafish

## Abstract

Telomere length, unlike most genetic traits, is epigenetic, in the sense that it is not fully coded by the genome. Telomeres vary in length and randomly assort to the progeny leaving some individuals with longer and others with shorter telomeres. Telomerase activity counteracts this by extending telomeres in the germline and during embryogenesis but sizeable variances remain in telomere length. This effect is exacerbated by the absence of fully active telomerase. Telomerase heterozygous animals (*tert^+/−^*) have reduced telomerase activity and their telomeres fail to be elongated to wild-type average length, meaning that – with every generation – they decrease. After a given number of successive generations of telomerase-insufficient crosses, telomeres become critically short and cause organismal defects that, in humans, are known as telomere biology disorders. Importantly, these defects also occur in wild-type (*tert^+/+^*) animals derived from such *tert^+/−^* incrosses. Despite these *tert^+/+^* animals being proficient for telomerase, they have shorter than average telomere length and, although milder, develop phenotypes that are similar to those of telomerase mutants. Here, we discuss the impact of this phenomenon on human pathologies associated with telomere length, provide a brief overview of telomere biology across species and propose specific measures for working with telomerase-deficient zebrafish.

## Teaser

**Summary:** Telomere length can be considered an epigenetic trait that depends on the genetic telomerase status as well as on inherited progenitor telomere length. This can impact human disease for generations.

## Inheritance of telomere length and associated diseases

Telomeres, i.e. the ends of eukaryotic chromosomes, are replicated differently compared with the rest of the genome. Chromosome ends pose a special challenge to conventional DNA polymerases. Given the requirement of a template sequence to synthesise a new strand, the very end of the chromosome is incompletely duplicated and shortens with every round of cell division – known as the ‘end replication problem’. Pioneering work by Elizabeth Blackburn, Carol Greider and Jack Szostak, who were later awarded the Nobel prize, identified a specialised DNA polymerase, telomerase (*tert*), dedicated to elongating chromosome ends (Greider and Blackburn, 1985). Telomerase is a ribonucleoprotein polymerase complex that employs the RNA of one of its subunits, i.e. of telomerase RNA component (*terc*), as a template, adding repeated copies to the ends of chromosomes, thereby preventing replicative shortening (de Lange, 2006).

Telomere length is maintained from generation to generation by the action of telomerase, which elongates telomeres in the germline and during embryogenesis. However, in humans, telomerase expression is repressed in most somatic cells after birth, so telomeres shorten throughout our lives. This may have evolved as a tumour suppressor mechanism to limit harmful cell proliferation (Forsyth et al., 2002; Cong et al., 2002). As a trade-off, continuous telomere erosion eventually results in replicative senescence and contributes to ageing pathologies (Henriques and Ferreira, 2012; Pereira and Ferreira, 2013).

Even though telomerase expression during embryonic development maintains telomere length for generations (Zheng et al., 2014), telomeres do not have precise lengths. They vary between different chromosomes, and also between cells and tissues of the same organism. The primary cause is that telomerase does not elongate telomeres to an exact length (Shore and Bianchi, 2009). Telomerase acts primarily on short telomeres, going through cycles of synthesis until it disengages (Zheng et al., 2014; Zhao et al., 2011). Telomere elongations depend on telomerase activity and the regulatory role of the telomere-binding complex, known as shelterin. A second reason for telomere length variation across different tissues is the rate of cell division. The more frequently somatic cells divide, the faster telomeres shorten. As we age, this is observed in the gastrointestinal and hematopoietic systems (Lansdorp, 1995; Aubert et al., 2012; Demanelis et al., 2019), due to their high rates of turnover. A third reason for telomere variation is damage in response to environmental and cellular stress. Telomeres are G-rich sequences, i.e. they comprise a stretch of three or more continuous guanine residues, and thus are especially prone to oxidative damage (Hewitt et al., 2012; Fumagalli et al., 2012). This is particularly important in highly metabolic organs, such as the brain and heart, as they have increased levels of mitochondrial reactive oxygen species. In these cases, the protective nature of telomeric DNA leads to poor repair of the DNA lesion 8-oxoguanine. This is particularly pertinent in post-mitotic cells, such as neurons and muscle. Accumulation of damaged DNA at telomeres leads to rapid telomere attrition, beyond the expected gradual loss with cell division.

Given its diversity among individuals, average telomere length does not constitute a strong predictor of biological age. Telomere length is, however, associated with several pathologies, including COVID-19, lung, liver, hematologic and cardiovascular diseases, as well as multiple forms of cancer

Telomere length in the zygote is fixed by the gametes (Chiang et al., 2010). However, as in other cells, telomere length in gametes varies, despite the constitutive expression of telomerase in germ cells (Zvereva et al., 2010). Furthermore, the gradual reduction of telomerase activity and the many rounds of cell divisions involved in the zygote-to-embryo development all the way to adulthood, give rise to the diversity of telomere length present in our bodies. Telomere decline is fastest in our first years of age; this decline is still very rapid until our late teens, slowing down as we reach adulthood to a steady state turn over (Sidorov et al., 2004). Surprisingly, even though telomeres shorten regularly as we age, the diversity of telomere length among individuals is considerable, meaning that some young individuals have telomeres of the average length of those of old people and vice versa. This was observed in leukocytes harvested from hundreds of people across different ages (Aubert et al., 2012; Hou et al., 2015; Werner et al., 2015). Given its diversity among individuals, average telomere length does not constitute a strong predictor of biological age (Der et al., 2012). Telomere length is, however, associated with several pathologies, including COVID-19 (Mahmoodpoor et al., 2023), lung (Amsellem et al., 2011), liver (Wiemann et al., 2005), hematologic and cardiovascular diseases (Minamino and Komuro, 2007), as well as multiple forms of cancer (Campisi, 2013). Most studies found that these human diseases correlate with reduced leukocyte telomere length (LTL) (Byrjalsen et al., 2023; Raj et al., 2023). However, long telomeres can also be associated with cancer (Chen et al., 2023), most likely enabling escape from the replicative senescence barrier (Low and Tergaonkar, 2013). In addition to natural variation of telomere length and disease, mutations in the genes encoding telomerase subunits and its associated proteins, lead to syndromes called telomere biology disorders (TBDs) (Raj et al., 2023; Revy et al., 2023; Carvalho et al., 2022). These include rare diseases (Alter et al., 2012; Garofola et al., 2023), such as dyskeratosis congenita and Hoyeraal–Hreidarsson syndrome, but also chronic diseases, such as idiopathic pulmonary fibrosis (IPF) (Spagnolo and Lee, 2023). IPF affects ∼5 million people worldwide and half of them have telomeres with lengths in the lower 1% of the average distribution for their age group.

Telomere length is, therefore, an epigenetic trait that depends not only on the genetic status of telomerase but also on the telomere length that we inherit from our parents

Telomerase is dosage-sensitive and most TBDs result from heterozygosity of telomerase-associated proteins. Homozygous and compound heterozygous deficiencies are extremely rare and give rise to even shorter telomeres with severe phenotypes (Mason and Bessler, 2011). The fact that telomere length cannot be maintained by a single functional copy of telomerase is highlighted by the phenomenon known as genetic anticipation (Vulliamy et al., 2004; Armanios et al., 2005). Grandparents carrying a telomerase mutation in one allele – equivalent to generation 0 (G0) – might not have severe symptoms but the chances of phenotypes presenting does increase substantially in the subsequent generations of carriers. This happens for two main reasons. First, a heterozygous individual (G0) produces shorter telomere gametes and, second, the newly formed heterozygous zygote (G1) is unable to maintain the parental telomere length when undergoing the multiple cell divisions into adulthood. This telomere shortening is, thus, exacerbated in subsequent generations (G2 onwards). Progressive telomere shortening from generation to generation in heterozygosity was also shown in model systems, such as CAST/Ei mice (a strain derived from the wild *Mus musculus castaneus*) (Hathcock et al., 2002; Hao et al., 2005) and zebrafish (Scahill et al., 2017). The most striking result of this generational decline is that, upon successive heterozygous incrosses, genetically wild-type telomerase descendants with fully functional telomerase activity, also exhibit very short telomeres and phenotypes associated with the telomerase mutation (Hao et al., 2005). Telomere length is, therefore, an epigenetic trait that depends not only on the genetic status of telomerase but also on the telomere length that we inherit from our parents (Zheng et al., 2014; Njajou et al., 2007).

## Laboratory models to investigate the consequences of telomere shortening

Reduced telomere length and restriction of telomerase expression appear to have evolved in response to different life strategies across species. For example, lifespan and telomere length of killifish are inversely correlated in the wild (Reichard et al., 2022), as short-lived killifish strains from drier climates possess longer telomeres than longer living strains. Telomere length also inversely correlates with lifespan in mammals (Gomes et al., 2010, 2011). Large mammals, which undergo more cell divisions and live longer, have evolved tumour suppressor mechanisms that rely on cell division clocks, such as telomere shortening. Also, male killifish, which are overall larger than females, have shorter telomeres (Reichard et al., 2022). Interestingly, the naked mole rat, which – despite its small size – lives for up to 30 years, has shorter telomeres than most of its closely related rodents that have shorter lifespans (Gorbunova and Seluanov, 2009). Laboratory mouse strains (e.g. C57BL/6, BALB/c, etc.) mostly derived from *Mus musculus domesticus* have 5 to 10 times longer telomeres than humans and have largely telomerase-independent cell division counting mechanisms (Forsyth et al., 2002; Wright and Shay, 2000; Itahana et al., 2004). G1 telomerase-deficient (*Tert^−/−^*) lab mice retain very long telomeres and do not display decreased fertility, survival or increased disease associated with decreased telomere length. Nevertheless, telomere dysfunction does, eventually, impact mouse health because *Tert^−/−^* mice obtained through several generations of incrossing (G4–G6) develop age-related pathologies (Blasco et al., 1997; Lee et al., 1998; Rudolph et al., 1999).

Zebrafish age-dependent tissue degeneration occurs in a time- and tissue-dependent manner […] this is reminiscent of the human scenario, where telomerase loss-of-function mutations or mutations affecting telomere stability lead to premature ageing syndromes […] with particular impact on highly proliferative tissues

Zebrafish (*Danio rerio*) recently emerged as a powerful complementary model to investigate the fundamental mechanisms of ageing that underlie disease (Carneiro et al., 2016a). Zebrafish display various age-associated phenotypes that mimic those of human ageing, including spine curvature (Henriques et al., 2013; Anchelin et al., 2013; Gerhard et al., 2002), retinal atrophy and cataracts (Carneiro et al., 2016b; Anchelin et al., 2013; El Mai et al., 2023), infections (Carneiro et al., 2016b), loss of body mass (wasting) (Carneiro et al., 2016b; Trueland, 2013; Takahashi et al., 2017; Gerhard et al., 2002), cancer (Carneiro et al., 2016b), neurodegeneration (Kishi et al., 2008) and altered behaviour (Espigares et al., 2021). Importantly, critically short telomeres in zebrafish are associated with increased DNA damage response (DDR) markers, and the accumulation of DDRs predicts increased cellular senescence and age-associated pathology (Carneiro et al., 2016b). Many of these age-associated changes are accelerated in the absence of telomerase, allowing the distinction between telomerase-dependent and -independent phenotypes (Carneiro et al., 2016b; Henriques et al., 2013). In G1 adult *tert^−/−^* zebrafish, progressive shortening of telomeres, accumulation of senescence and inflammation occurs over a relatively short period of time, i.e. between 6 and 12 months as compared to between 24 and 36 months in wild type, allowing an exceptional temporal analysis from young to old *tert^−/−^* animals (Carneiro et al., 2016b). Zebrafish age-dependent tissue degeneration occurs in a time- and tissue-dependent manner, with highly proliferative tissues, such as the intestine and blood, amongst the first to be affected. Importantly, this is reminiscent of the human scenario, where telomerase loss-of-function mutations or mutations affecting telomere stability lead to premature ageing syndromes – such as TBDs or, in extreme cases, progerias – with particular impact on highly proliferative tissues, like the gut (Alter et al., 2012; Hofer et al., 2005).

**Fig. 1.**
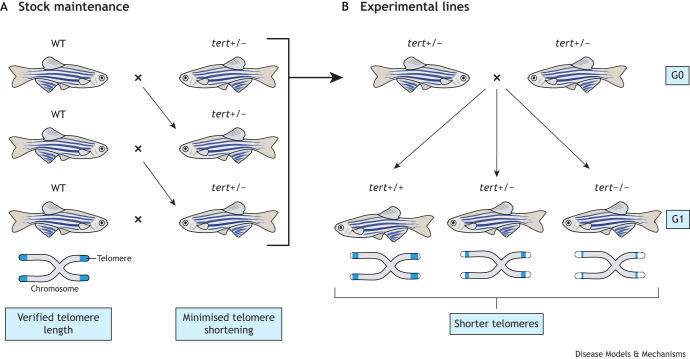
**Maintaining stocks and experimental lines of telomerase mutants.** (A) Breeding stocks of *tert^+/−^* zebrafish must be generated by outcrosses to wild-type (WT; *tert*^+/+^) fish. WT zebrafish used for this should be from a stock with verified telomere length. The *tert*^+/−^ progeny can be used in consecutive crosses for stock maintenance, as outbreeding with the WT stocks introduces new ‘pools’ of telomeres with diverse length. This minimises (but does not completely eliminate) telomere shortening across generations. All generations of *tert*^+/−^ zebrafish from stock maintenance can be used to produce experimental lines, but the age of the parents used must be consistent. (B) Crossing *tert*^+/−^ zebrafish generated from stock maintenance (G0) will produce *tert*^+/+^*, tert*^+/−^ *and tert*^−/−^ progeny (G1). Owing to haploinsufficiency, G0 *tert*^+/−^ zebrafish will have reduced capacity to extend telomeres, so G1 *tert*^+/−^ as well as *tert*^+/+^ zebrafish will inherit shorter telomeres (indicated by dotted lines), with G1 *tert*^+/−^ zygotes having reduced capacity to extend their telomeres. Therefore, *tert*^+/−^ zebrafish incrosses must not be used for stock maintenance. As *tert*^−/−^ zebrafish completely lack telomerase, they will have even shorter telomeres and associated phenotypes will be apparent in the first generation. G0, generation 0; G1, generation 1; tert, telomerase reverse transcriptase; WT, wild type.

Like in mice and humans, telomerase expression is haploinsufficient in zebrafish. Similar to *tert^−/−^* mutants, descendants of older (16-month-old) heterozygous (*tert^+/−^*) fish have shorter telomeres and show more prominent signs of cachexia and fertility problems compared to descendants of younger (4-month-old) *tert^+/−^* parents (Scahill et al., 2017). This suggests that *tert* haploinsufficiency manifests in gametogenesis and progressively worsens as parents age, due to further telomere shortening throughout life. It is worth remembering that in humans, oogenesis only occurs during embryonic development but spermatogenesis continues until much later in life, which impacts how these findings are translated from zebrafish. Furthermore, with every generation of successive *tert^+/−^* incrossing, the phenotypes become progressively more severe. For instance, body wasting and early infertility are apparent in early generations of *tert^−/−^* progeny (G1), but this is anticipated in *tert^+/−^* and *tert^+/+^* zebrafish after multiple incrosses of *tert^+/−^* zebrafish, rendering the line non-reproductive. We, therefore, have recommendations to avoid unintended haploinsufficiency when using zebrafish (Box 1 and Fig. 1).

Thus, not all organisms rely on telomere shortening during their lifetime as a mechanism to prevent disease and decline of fitness. For this reason, it is vital to seek out the appropriate model system to answer the questions at hand. Sometimes, the closest evolutionary model is not the most relevant.

**BOX 1: Recommendations to avoid unintended haploinsufficiency effects in zebrafish**

**1 -** To reduce the effects of variability in telomere length and haploinsufficiency, compare telomerase-deficient animals with WT siblings derived from a G1 heterozygous cross. In addition, use animals of identical age and keep the same couples to generate siblings (e.g. three different couples to create the progeny). In zebrafish, *tert^−/−^* (allele hu3430) animals are infertile when aged 6 months or older (Henriques et al., 2013; Anchelin et al., 2013). It is crucial for breeding stocks of this allele to be maintained in heterozygosity (*tert^+/−^)* generated by outcrosses to WT, i.e. *tert^+/−^× tert^+/+^* (see Fig. 1).

**2 -** Telomere length of WT stocks also matters. As an example, given the outbred nature of zebrafish, telomere length varies (∼2-fold) across strains and should be verified before choosing the *tert^+/+^* stock line. Introgressing the telomerase mutation into a different genetic background can modify ‘baseline’ telomere length and, therefore, timing of critical decline in telomere length.

**3 -** In telomerase mutant animals generated by chemical mutagenesis, such as in the well-described zebrafish (tert^hu3430^), the original line was outcrossed to AB WT animals at least five times to minimise the possibility of other non-related mutations. This is particularly important for zebrafish lines available at the Zebrafish International Resource Center, generated by ENU mutagenesis and which have not yet been outcrossed. Of note, with the exception of the hu3430 strain, other zebrafish telomerase mutants (*tert ^sa6541^* and *tert ^sa25076^*) (Scahill et al., 2017) have not yet been assessed for telomerase activity and, thus, not yet shown to be complete telomerase-deficient animals.

**4 -** Although effort has been made to harmonise the feeding protocols across zebrafish facilities, it is still the case that most have different regimens with varying nutritional values. Caloric restriction and/or high-fat diets, known to alter lifespan in many models, are likely to interfere with results obtained in different labs. Information regarding consistency concerning the number of feeds per day, feeding on weekends and usage of live prey (e.g. artemia and rotifers) is widely variable. This is particularly important when performing long-term experiments aiming to investigate longevity, neurodegeneration and tumour incidence. Thus, declaring the feeding regimen and harmonising with other animal facilities is of utmost importance.

**5 -** Telomere shortening rates differ across tissues. Importantly, in zebrafish, gut-specific rescue of the telomerase mutant results in a time-dependent improvement of the whole organism, including fertility and longevity (El Mai et al., 2023). Therefore, effects of telomerase mutants can be non-autonomous and should be considered when performing phenotypic analysis in particular tissues.

**Figure:**
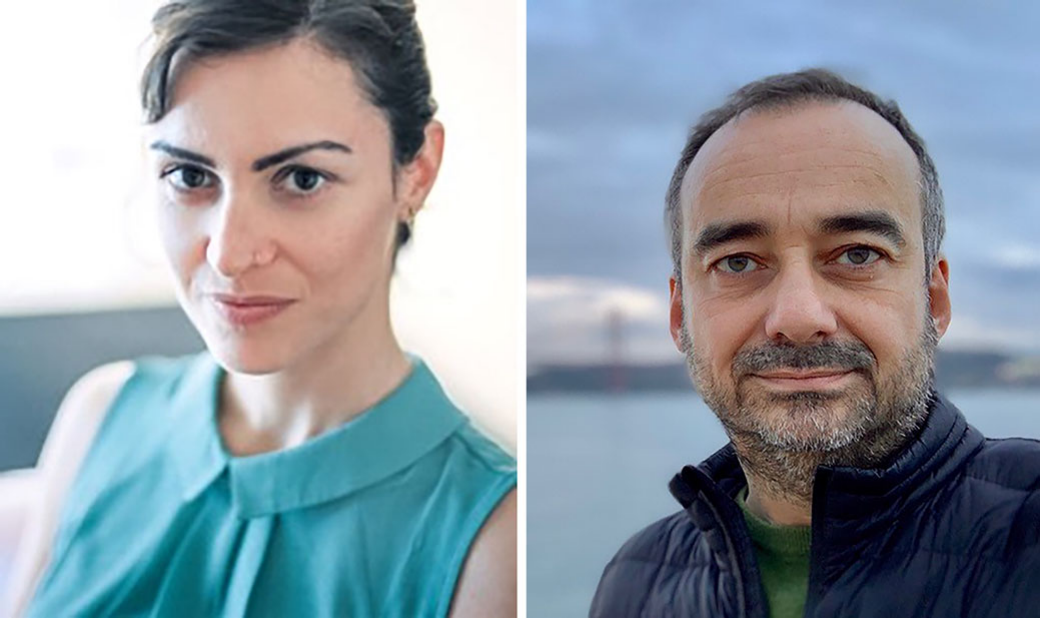
Catarina M. Henriques (left) and Miguel Godinho Ferreira (right)

There are other model systems in which telomere shortening also causes organismal functional decline across multiple generations. Examples include ciliates and yeast (de Lange, 2006), plants (Riha et al., 2001), *Caenorhabditis elegans* (Meier et al., 2006) and the aforementioned killifish (Harel et al., 2015). In these examples, apart from killifish, complete absence of telomerase does not result in telomere-associated phenotypes in G1. Similar to *Tert^−/−^* laboratory mice, G1 homozygous telomerase knockouts of *C. elegans* (Meier et al., 2006) and *Arabidopsis thaliana* (Riha et al., 2001), do not exhibit an observable impact on survival and reproduction. However, defects do occur when homozygous telomerase knockouts are incrossed for several generations, forcing telomere decline and genetic anticipation, as described for heterozygous deficiencies in humans and zebrafish. This contrasts with humans, zebrafish and killifish, in which telomerase deficiency results in severe phenotypes in the first mutant generation. Thus, not all organisms rely on telomere shortening during their lifetime as a mechanism to prevent disease and decline of fitness. For this reason, it is vital to seek out the appropriate model system to answer the questions at hand. Sometimes, the closest evolutionary model is not the most relevant.

## Conclusions

Different organisms display varying responses to telomere shortening, with some showing delayed effects over several generations. Understanding the diversity of telomere length and its evolutionary adaptive role in nature remains largely undetermined given the focus on human telomere biology. Exploring the diverse outcomes of telomere dysfunction in organisms as varied as zebrafish, wild mice and humans, will enhance our understanding of the role telomere shortening has in aging and disease. Telomere length, as an epigenetic trait, is dictated not only by expression of telomerase but also depends on the tissue context (e.g. replicative potential, oxidative stress, metabolism, etc.) and the surrounding environment. Exposure to stress from early development will condition adult telomere length and translate to increased susceptibility to disease and premature aging. A more thorough understanding of these processes across species will provide new avenues for telomere elongation strategies in TBDs and, potentially, other pathologies associated with shortened telomeres, thus, helping to establish a clear period of intervention while avoiding the ever-present risk of cancer.
